# Supplementary figures and images for: Effects of Silver Nanoparticles on Proliferation and Apoptosis in Granulosa Cells of Chicken Preovulatory Follicles: An In Vitro Study
Source: Animals (Basel). 2021 Jun 2;11(6):1652. doi: 10.3390/ani11061652 (PMC8229578; doi:10.3390/ani11061652)

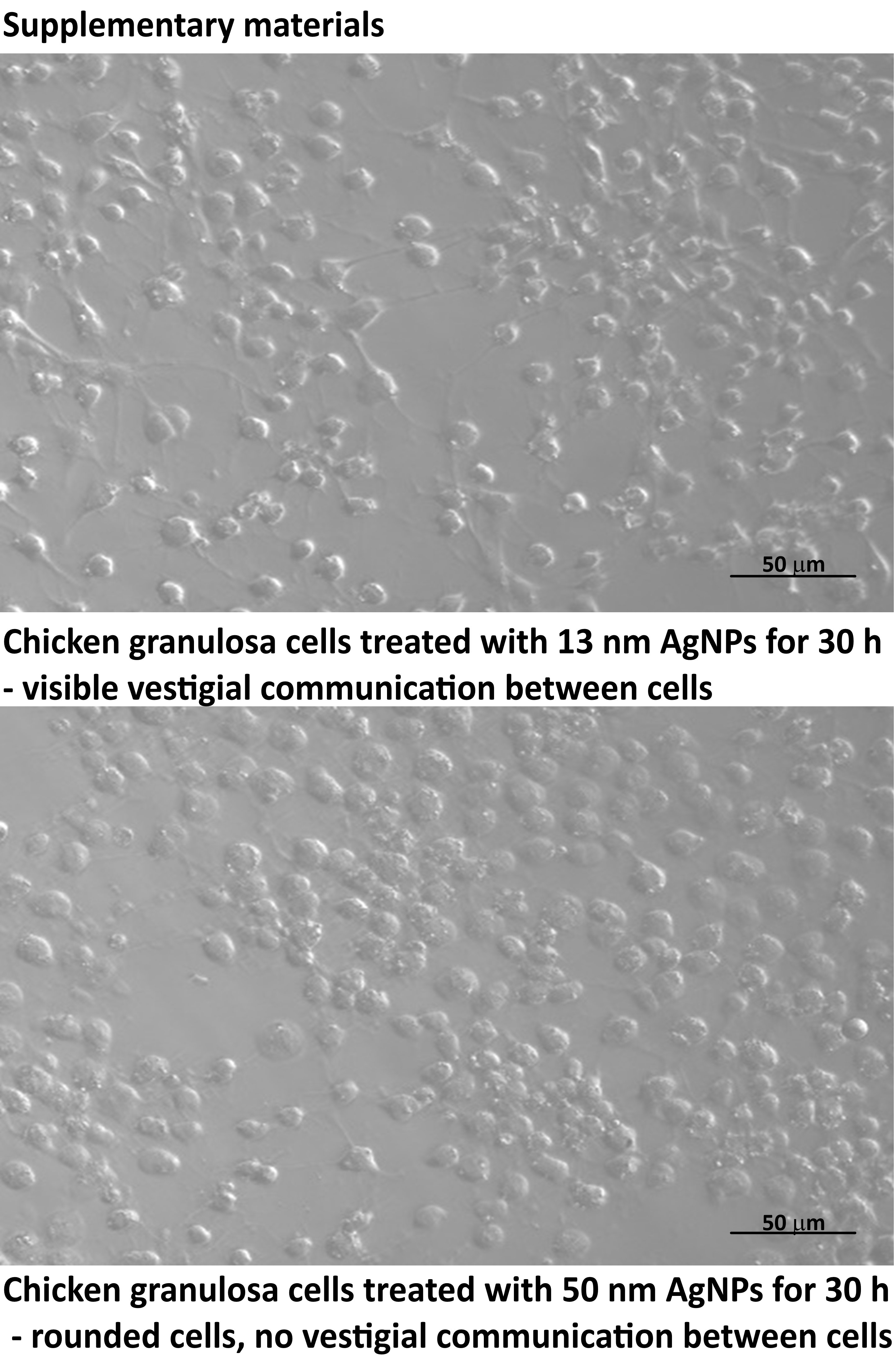

Supplement: Supplementary file 1 [file animals-11-01652-s001.zip › animals-1237102-supplementary.tif]
